# Diving into Relevance: How Deep Sea Researchers Articulate Societal Relevance within their Epistemic Living Spaces

**DOI:** 10.1007/s11024-025-09577-z

**Published:** 2025-03-25

**Authors:** Sarah Rose Bieszczad, Maximilian Fochler, Sarah de Rijcke

**Affiliations:** 1 Centre for Science and Technology Studies, Leiden University, Leiden, The Netherlands; 2 Department of Science and Technology Studies, University of Vienna, Vienna, Austria; 3 Centre for Technology, Innovation and Culture, University of Oslo, Oslo, Norway

**Keywords:** Societal relevance, Articulations of relevance, Epistemic living spaces, Epistemic commitments, Deep sea

## Abstract

The deep sea is many things: a subject of both fascination and indifference; a realm of great unknowns and scientific inquiry; and a source of untapped potential and looming exploitation. Its multifaceted nature is also visible in scientific research on the deep sea. While traditional relevance narratives in the field of deep sea research centred around fundamental knowledge creation, calls for doing societally relevant research are intensifying. The field is currently in transition regarding its understanding of societally relevant research, as it has been criticised for failing to provide narratives that adequately demonstrate its societal relevance and the corresponding disconnect between its epistemic foci and the concerns of researchers, industry, and society more broadly. This transition is reverberating throughout ocean research institutions, prompting deep sea researchers to reconsider and re-articulate the relevance and applicability of their work. This article examines how deep sea researchers from two European ocean research institutes articulate the societal relevance of their work within their unique epistemic environments. Using a person-centred approach, we identify three ideal-type articulations of relevance: fundamental, translational, and solution-oriented. These articulations are shaped by financial, institutional, cultural, and media-related factors within the researchers' epistemic living spaces as well as their epistemic commitments to the creation of particular types of knowledge. Our findings reveal diverse understandings of societal relevance among deep sea researchers, even within single institutions. By focusing on these articulations, we frame relevance as an active process, highlighting the various actions researchers undertake to produce knowledge they perceive as relevant.

## Introduction^1^

The deep sea is many things: a subject of both fascination and indifference; a realm of great unknowns and scientific inquiry; and a source of untapped potential and looming exploitation. Deep sea research has a long history of fundamental research, with most of its justifications for doing research drawing heavily from narratives of exploration and the acquisition of knowledge of the vast unknown (Helmreich 2009; Adler 2019), often being far from societal concerns (Jamieson et al. 2021). Since the postwar period, the deep sea has been also a site for military technological development (see Oreskes 2021b). Still, the deep sea remains in large parts unexplored and understudied (Levin et al. 2019; Webb et al. 2010) and is generally considered as being further from society’s mind (Jamieson et al. 2021), particularly in comparison to coastal regions, which are more closely linked to societal concerns (Rogers et al. 2015).

However, over the past decade, the deep sea has come to be understood as a critical actor in the environmental cycles of the Earth system and as a reservoir of (extractable) valuable resources. This expansion of the perceptions of the deep sea coincides with both the increasing awareness of the climate crisis and the increasing economisation of the ocean across many fields in ocean research (Asdal and Huse 2023). This awareness corresponds to shifts in science policy towards valuing societally relevant research, particularly those which address grand societal challenges such as climate change and the transition to green energy. Ocean research is implicated in these transformations through the grand societal challenges formulated in the United Nations Decade of Ocean Science and the multiple subsequent science policy initiatives that followed, including the European Union’s Mission Area Restore Our Ocean and Waters and the UN’s Sustainable Development Goal (SDG) 14: Life Below Water. These transformations incentivise collaborations with industry, particularly for finding innovative solutions to grand societal challenges. For example, deep sea mining has been proposed as one solution to obtaining enough rare-earth elements necessary for the green energy transition and the energy sector is eager to collaborate with ocean research to study this solution. However, many potential impacts of mining on the deep sea environments remain unknown, which creates tensions between environmental protection and exploitation.

Ocean research institutes and deep sea researchers alike are facing significant changes as societally relevant research begins to take centre stage. Until recently, deep sea research was primarily focused on fundamental research lines and relied on basic or excellence funding, especially in European public research institutions.^2^ The deep sea is notoriously hard to access and the requirements for conducting research there (e.g., accessing the deep sea and securing mediating technologies) are resource- and infrastructure-intensive. Thus, institutions and researchers engaging in deep sea research need to aggregate large amounts of funding to ensure the continuation of their research (Oreskes 2021a; Rogers et al. 2015; Bell et al. 2023). Consequently, researchers and institutions alike need to be responsive to changes in funding policies and environments, such as calls for closer collaborations with industry partners (Howell et al. 2020).

Given its history and current role in addressing societal challenges, deep sea research is an interesting case to study how researchers come to understand their research as societally relevant and to explore how their (potentially) changing understandings relate to larger shifts in science policy and research towards valuing societally relevant research. Drives for a more responsive science are increasingly framing research and innovation policy directives (Ulnicane 2016; Mazzucato 2018), as well as the policies and practices for incentivising and evaluating their accompanying transitions. Concurrently, well-established regimes of worth with evaluative principles such as research excellence, competitiveness, or productivity, co-exist and sometimes conflict with emerging regimes and principles, including innovativeness and relevance (Falkenberg 2021; Pinel 2021; Rushforth et al. 2019). These co-existing regimes are now coinciding with policy and research governance strategies pushing for - and potentially generating new regimes of - (societal) relevance, creating potential tensions for both researchers and their research institutions, who must navigate and adjust accordingly to these multiple, coexistent regimes (Rushforth et al. 2019; Granjou and Arpin 2015; Kaltenbrunner 2020).

As the term societal relevance expands in use, its definitions and articulations remain heterogeneous and often context and field-specific (Hessels et al. 2009). Moreover, like the term societal relevance, deep sea research is also diverse. To attend to the diversity of both, this article focuses on societal relevance as *the perceived worth research can acquire in relation to society*. This definition allows space for researchers to construct their own understandings of the societal relevance of their research without any a priori normative categories of what constitutes ‘good’ or ‘relevant’ research. This article takes the case of deep sea research as a focal point for exploring current shifts towards valuing societal relevance. We will examine how and in what ways deep sea researchers come to understand and articulate the worth of their research in relation to society.

While deep sea research is still in the process of incorporating more societally relevant research lines and collaborations (Rogers et al. 2015), previous studies in Science and Technology Studies (STS), in contrast, considered articulations of relevance within fields with more directed knowledge production practices. Some examples are biodiversity studies, which arose from specific environmental concerns (Granjou and Arpin 2015), and soil science, which arose as application-based science and had a legitimisation crisis that fostered re-articulations to regain societal relevance (Sigl et al. 2023). The historical and present relation to societally relevant research in deep sea research creates an interesting opportunity to investigate epistemic commitments in articulations of relevance within a very different epistemic and disciplinary context.

Using a person-centred approach across two institutional contexts, we investigate how deep sea researchers articulate the societal relevance of their research within their epistemic living spaces (Felt 2009), which attend to “the efforts of researchers to stabilise, extend or protect the space they occupy socially and epistemically, as well as institutionally” (Felt and Fochler 2012, 5). In doing so, we identify three articulations of societal relevance adhered to by deep sea researchers when narrating the worth of their research in relation to society. We describe how these articulations are shaped by the researchers’ commitments to the creation of certain types of knowledge within the larger ecology of their epistemic living spaces, which we analyse across four features of epistemic living spaces: institutional context, financial and evaluative factors, cultural norms, and mediatisation (ibid). We will also show the influence that epistemic living spaces have on researchers’ epistemic commitments (Granjou and Arpin 2015; Sigl et al. 2023). As we will show, the actions researchers take to remain aligned with these commitments depend on and are informed by the epistemic living spaces they inhabit. Through our three articulations of societal relevance in deep sea research, we demonstrate that there is no single way in which researchers conceptualise their research as relevant. We conclude that there is no one-size-fits-all method for incentivising research(ers) in finding solutions for grand societal challenges.

### Articulating Societal Relevance in Epistemic Living Spaces of Deep Sea Research

Reflective of wider transitions in the science system from mode one to mode two knowledge production (Nowotny et al. 2001), understandings of societal relevance are also broadening and transitioning.^3^ In mode one, societal relevance is seen as external to the knowledge production process, following the classic linear model of innovation wherein the results of basic research are taken up by external actors for societal use. In contrast, within mode two, relevance helps shape the knowledge production process within science; thus, attuning it more closely to societal needs. As the concept of societally relevant research expands, more scientific fields are expected (if not obligated) to contribute to addressing societal goals. The expanding use of the term relevance corresponds to a growing multiplicity of meanings, applications, and expressions across a variety of scientific disciplines and their respective practices.

Still, Hessels et al. (2009, 388) succinctly define the term as “the expected value scientific research will have for society.” They also call for more research on how and in which contexts science is made relevant for societal issues and the consequences thereof for research and policy. Recent studies demonstrate the tensions that arise when pushes for relevance co-exist with other forms of evaluative principles in research, such as research excellence (Bandola-Gill 2019), or when coping strategies for reconciling multiple understandings of relevance across disciplinary boundaries are limited (Schikowitz 2020). Furthermore, these tensions are exacerbated when researchers and disciplines (re-)articulate the relevance of their research in diverse ways (cf. Granjou and Arpin 2015; Sigl et al. 2023), often creating frictions with other pre-existing academic and evaluative structures, such as institutional settings, career requirements, conflicting regimes of worth, and funding structures (Falkenberg 2021; Kaltenbrunner 2020; Rushforth et al. 2019; Fochler et al. 2016; Felt 2017; Sigl et al. 2020; Pinel 2021).

Building on Hessels et al. (2009), we adopt a more situated, present-and process-oriented definition of relevance as *the perceived worth research can acquire in relation to society.* In doing so, we examine how researchers themselves articulate the relevance of their work from their own situated perspectives in the present. Our use of the term articulation borrows from van Lente and van Til’s (2008) use of the term, which focuses on the narrative process wherein broad terms (such as societal relevance) are specified and legitimised in relation to one’s pre-existing frameworks and values. We expand on their use by understanding articulations of societal relevance as narrative expressions of the relations between researchers’ understandings of their epistemic work and the contexts that they inhabit (e.g., institutional, funding, etc.). We conceptualise these contexts as their epistemic living spaces, or their “individual or collective perceptions and narrative re-constructions of the structures, contexts, rationales, actors, and values which mould, guide, and delimit their potential actions” (Felt and Fochler 2012, 5). Epistemic living spaces adopt a person-centred approach to tease out the specific contexts, values, and valuations from which researchers draw whilst articulating the relevance of their research. While Felt and Fochler (2012) identify seven features of epistemic living spaces, our analysis focuses on four: institutional context, financial and evaluative factors, cultural norms, and mediatisation. As we will show, these four features deeply shape deep sea researchers’ articulations of societal relevance.

Valuation studies scholars have had a particular interest in examining the values and valuations behind attributing worth to a certain idea or situation (cf. Lamont 2012; Heuts and Mol 2013), including the worth of research for society. A subset of this literature has focused on valuations in an academic setting. Fochler et al. (2016, 180) introduce regimes of valuation as “the broader discursive, material, and institutional background” that concrete evaluations draw on. Building on this, Rushforth et al. (2019, 6) define regimes of worth as “evaluative principles and associated forms of valuation that become stabilised in institutional settings to the point where they constitute an obligatory framework that individuals and groups must accommodate in their research”. STS scholars use these terms to better understand how researchers live with multiple evaluative principles and regimes within contemporary academia (Fochler et al. 2016; Schikowitz 2020; Felt 2017; 2009), including relevance (Rushforth et al. 2019; Falkenberg 2021; Kaltenbrunner 2020; Sigl et al. 2023; Granjou and Arpin 2015). These studies show: how researchers attempt to incorporate new regimes of worth, which often clash with other regimes of worth that are important to researchers’ practices (Falkenberg 2021); how they curate research portfolios to appeal to and take advantage of multiple regimes of worth to ensure multiple funding streams (Rushforth et al. 2019); and how they attempt to reduce uncertainty when confronted with newer regimes of worth (Kaltenbrunner 2020).

Linking valuations of relevant research and how relevance is (re)articulated, Granjou and Arpin (2015) propose the term epistemic commitments, or the “ethical and political commitment to the production of a certain type of knowledge” (1025). They identify epistemic commitments in biodiversity; a field that since its inception, has been interwoven with environmental governance and political action, leading to a wide array of research approaches, collaborations, and concerns. Thus, the field was well placed for Granjou and Arpin (2015) to show how four identified regimes of research each correlate to their own approaches, technologies, scenarios, and practical contributions to which researchers become committed. Building on their work, Sigl et al. (2023) explore how the discipline of soil science renegotiated its relevance and self-understanding. They identified five epistemic commitments, which correspond to re-articulations of relevance. The authors expand on Granjou and Arpin’s work by demonstrating how epistemic commitments and articulations of relevance change over time, as well as how they are (re)negotiated in relation to other forms of worth in academia.

The studies discussed above have made important conceptual inroads into (re)articulations of relevance and their corresponding epistemic commitments, but the empirical cases in which articulations of relevance have been studied remain limited. While Granjou and Arpin (2015) and Sigl et al. (2023) targeted field-level changes in articulations, we study this variation on a much smaller scale: in two institutional contexts. In doing so, we show that a multiplicity of articulations of relevance is not merely an exclusive characteristic of large scales, but can even be found within research institutions, across the hallway, among colleagues.

### A Brief History of Deep Sea Research

Human understanding of the deep sea has evolved majorly over time. In the early 19th century, as contemporary naturalists began to theorise about the deep scientifically, the general understanding of the ocean’s depths transformed from a mysterious place with dangerous, mythical creatures to a static, barren barrier to be crossed (Helmreich 2009). Around the end of the 19th century, the invention and the necessity of emerging technologies began to alter the ways both science and society conceptualised the deep sea. After the invention of sonar and the instalment of underwater trans-oceanic telecommunication cables, the deep sea began to be conceptualised in three dimensions and understood as containing both familiar and novel forms of life (Helmreich 2009; Starosielski 2015). Still, it was seen as unendingly vast and disconnected from human concerns and influences.

Research in deeper parts of the ocean began in earnest only in the early 20th century (Adler 2019; Rozwadowski 2005). Through the work of ocean-going naturalists (who aimed to expand the range and capability of their research), a framework was put in place for researching the deep sea to create standardised procedures and tools. These early researchers worked on ships commissioned for commercial or military purposes, which created certain well-studied routes and left vast parts of the ocean unknown (Oreskes 2021a). This correlation between a heavy reliance on funding and opportunistic approaches to data collection is still ongoing in present-day deep sea research. However, due to the expense and scale of the research (i.e., large-scale observation requiring multiple expeditions), it quickly shifted to institutions to ensure continual funding and support from nation-states. In the mid to late 20th century, scientists began to understand that the deep sea is full of life, highly dynamic, and a driving force across many Earth systems, e.g., physical, biological, and social (Oreskes 2021a). This understanding advanced alongside the mediating technologies necessary to explore the ocean’s depths. These advancements were largely due to increased funding during the Cold War period (Oreskes 2021b). Yet, despite these new insights, the ocean was still considered unendingly vast, with research during this time focused heavily on discovering unknowns. Scientists were only able to show that human activities were altering the entire ocean, including the deep sea, at the beginning of the 21st century (Oreskes 2021a). This is when they came to see that the ocean and its resources were in fact finite. The fact that the deep sea plays a central role in the Earth’s climate regulation and is a much-needed resource for obtaining rare elements as well as a place for carbon storage are recent insights—making it a key interest for policy as societies try to deal with multiple global crises.

Not unlike their predecessors, deep sea researchers today need to secure large amounts of funding, gain access to limited infrastructure, and foster strong collaboration networks (Rogers et al. 2015; Bell et al. 2023). The traditional relevance narratives of deep sea research also still centre around its novelty and the production of fundamental knowledge. These narratives not only often fail to demonstrate the relevance of the deep sea for society (Jamieson et al. 2021) but also are reflective of a larger disconnect between epistemic foci and concerns of scientific researchers and industry (Rogers et al. 2015). The importance of deep sea research in these justifications is placed on exploration and discovering unknowns and not on the significance of the deep sea for the Earth system and industry, creating tensions.

More and more the deep sea is being discussed by policy and industry actors as a potential well of resources such as oil, rare earth metals, and novel compounds (Thurber et al. 2014), with one study by the UN estimating the market value of the deep sea around 423 billion USD (Ottaviani 2020). These discussions are in part prompted because more applied uses of the deep sea are currently in the process of becoming both technically and economically feasible (Rogers et al. 2015). Nevertheless, there remain tensions in deep sea research between older legitimation narratives around fundamental research and rapidly changing and intensifying interests in the deep sea, both as a player in grand societal changes and a valuable resource for industry. How deep sea research, research institutes, and individual researchers incorporate these changing and expanding conceptualisations of the role(s) of the deep sea is still developing with many unknowns.

## Research Approach and Methods

The research for this article draws from the wider project *FluidKnowledge: how evaluation shapes ocean science*, which investigates how changing research evaluation systems and science policy efforts shape ocean science across national and institutional contexts. The material for this article was collected as part of a sub-project focusing on deep sea research and how this broad, interdisciplinary community of researchers align their epistemic agendas with their current situated academic realities.

The article draws from a range of empirical material collected through ethnographically informed interactions with two ocean research institutions in two national contexts that do research in and on the deep sea: one in Germany (Institute A) and one in the Netherlands (Institute B). Engagement with both research institutions was iterative, with initial online engagements occurring in the winter of 2021 at Institute A and spring of 2022 at Institute B, and in-person interactions beginning in the fall 2022 at Institute B. Institute A is an ocean research institute in Germany, which has a focus on the deep sea and (sub)seafloor. Most of the research conducted at Institute A is on deep sea topics, with only a few strands of research on shelf and coastal regions. The institute enjoys strong base funding and has received a German Cluster of Excellence (running for seven years), which focuses much of the institute’s attention on fundamental deep sea research. During our engagement with Institute A, the institute was preparing for another excellence cluster application that would determine if this prestigious and sizable funding stream would continue for another seven years. In the previous successful cluster application, the institute purposefully focused solely on basic research. According to the institute’s director, the application’s main critique in the review process was not including any societally relevant research, something to be adjusted for in the next application. At the time of the engagement, Institute A was also in the early stages of an active transitional period prompted by institutional governing bodies (their central board and general assembly), to try and incorporate more societally relevant research streams. This created greater reflection amongst its researchers about making their research more relevant and generated a unique instance to study ongoing articulations of societal relevance.

In contrast to Institute A, Institute B in the Netherlands has a wide range of epistemic foci, and its deep sea research is limited to one department. Additionally, Institute B faces more austerity in the context of the Dutch research funding landscape. This austerity is due to a combination of factors ranging from budget cuts in national funding bodies to an increasingly conservative government that actively pushes for funding to be directed towards the interests of industry and business. Still, many researchers at this institute are very highly performing in terms of obtaining external grants in both excellence research streams (European Research Council (ERC), Dutch National Research Council (NWO)) as well as more applied ones. For example, one large deep sea mining project was funded by an intergovernmental platform aiming to increase the efficiency and impact of research and innovation for sustainable, healthy, and productive seas and oceans. The Institute’s motto is “fundamental and applied frontier sea research,” which while showing its focus on fundamental research also shows its willingness to take up applied research lines. This sentiment is echoed by the institute’s director and apparent in their strategic agenda, which describes their research as “addressing important scientific and societal questions pertinent to the functioning of our oceans and our coasts to ensure a sustainable future.” Institute B sees itself as the leading fundamental ocean research institute in the Netherlands, but also responds to some calls for long-term environmental assessments (such as monitoring the subsidence of the Wadden Sea caused by oil extraction) and industry collaboration (such as assessing the impacts of windfarms and deep sea mining). Additionally, unlike Institute A, Institute B’s researchers tend to engage more heavily with research governance and policy bodies.

The main material for this paper comes from 22 semi-structured, qualitative interviews (Silverman 2015), with 12 researchers from Institute A and 10 from Institute B. The interviews were meant to create space for the researchers to openly discuss and reflect upon their career trajectories thus far and their current positions both in their field and institution, as well as consider what goes into developing their research portfolio and in which ways societal relevance of their research came into play. Supplementing the interviews are 12 participant observations (monthly institutional project meetings and quarter-annual board meetings at Institute A), six fieldwork days at Institute B (fieldnotes and participant observations) and an analysis of 32 supplemental documents from both institutes, ranging from strategic plans to various institutional meeting summaries. Additionally, the online presence and CVs of the deep sea researchers interviewed were analysed for career and topical trajectories. The series of 22 interviews ranged from 1.5 to 2.5 hours in length and were with researchers of various post-PhD career stages and paths. Their main disciplines ranged from deep sea biology (investigating deep sea vent environments, the deep biosphere, extreme environments, etc.) and deep sea chemical and physical oceanography (modelling and observing the currents, salinity, acidity, amongst other physical properties of the ocean and climate), to paleoceanography (investigating past ocean climates spanning millions of years) and marine geology and sedimentology (observing and modelling geological formations and sediment transfers). Some more applied research topics included deep sea mining, decommissioning oil, carbon storage in the deep sea, microplastics, ocean acidification, climate change, and spreading awareness of sand exploitation for building materials.

The first author developed and conducted the interviews and participant observation as well as collected other data for this article, which was anonymised to the best of our abilities taking into consideration what details provide texture to the argument. The semi-structured interviews were standardised using a (non-exhaustive) interview guideline to ensure consistency throughout the interviewing process, while also allowing space for adaptation and flexibility to facilitate the interviewee and their narrations. The interviews addressed the individual researchers’ narratives on the worth of their research for society within their epistemic living spaces, with the multiple sites accentuating institutional features that would remain invisible in a study of a single institutional context. The features include institutional context, financial and evaluative factors, cultural norms, and mediatisation. The collected data were analysed through a grounded theory-based approach utilising open coding (Charmaz 2006) followed by a thematic coding approach (Rivas 2018), which was performed using Atlas.ti. The first author analysed the interviews and supplemented the analysis with field notes from participant observations and accompanying documents. The interviews were first coded with a more open coding approach to create space for the individual interviewee and their narrations of the worth of their research in relation to society without preconceived definitions or indented topics. Thematic analysis was then applied iteratively to systematically tease out varying articulations of doing societally relevant research.

## Articulating Relevance in Deep Sea Research’s Epistemic Living Spaces

In this section we will describe three different articulations of societal relevance in deep sea research adhered to by researchers as they narrate the worth of their research in relation to society within their epistemic living spaces. It is important to note epistemic living spaces are co-constituted by many factors. They are not orderly or clearly defined spaces. Instead, they are highly complex and diverse, and continuously evolving by nature (Felt 2009). This inherent complexity is hard to capture in its entirety. Therefore, our three articulations are idealisations in such that they represent “ideal types,” which we employ to highlight the main differences between researchers’ understandings of the worth of their research in relation to society. Nevertheless, they exist as idealisations precisely because they are based upon major distinctions teased out of the “real world” messiness of the epistemic living spaces present in our data. We will revisit this point and illustrate it with examples in the discussion section.

To analyse the formation of articulations of relevance, we divide epistemic living spaces by four features institutional context, financial and evaluative factors, cultural norms, and mediatisation (Felt and Fochler 2012). Narrowing down to these features focuses our analysis and teases out influential elements in the shaping of these articulations. The institutional context plays a critical role, as institutional culture fosters certain norms, e.g., valuing excellence, while simultaneously not creating space for others, e.g., engaging with society. Financial and evaluative factors play a strong role in shaping how researchers argued for and adhered to certain epistemic commitments and understandings of relevance. Researchers would for instance add certain relevant research lines to their research portfolios to increase or sustain their chances of acquiring funding. Mediatisation, which we understand as communication with social actors, was a crucial issue in how researchers conceptualised the societal relevance of their work.

The sections that follow organise the articulations by their differences. The first major distinction, which separates the first articulation from the second and third, occurs between the types of knowledge the researchers understand as relevant for society and how this relevance comes to be. The first articulation’s relevance is centred around producing fundamental research about the earth system and it correlates fundamental knowledge directly with relevant knowledge. In contrast to the first articulation, the second and third articulations do not directly conflate societal relevance with the production of fundamental knowledge. Instead, these articulations recognise certain kinds of actions as necessary to make knowledge relevant. Our second major distinction, between the second and third articulation, arises in the type of actions required to make knowledge relevant, which differs depending on the researchers’ conceptualisations of science’s role in and relation to society.

### Articulation 1| Relevance as the Production of Fundamental Knowledge

This articulation centres societal relevance as *the creation of fundamental knowledge about the deep sea*, which is understood as inherently relevant itself, i.e., no further action is necessary to make fundamental knowledge relevant. Researchers adhering to this articulation narrate the deep sea as an integral part of the Earth system (e.g., climate cycling, sedimentation rates, understanding ocean currents, and biochemical processes occurring in natural environments) and, therefore, they see fundamental knowledge as inherently relevant to society. For example, when talking with a group leader in paleoceanography (the study of the past ocean) from Institute A about the societal relevance of their research, and their motivations for engaging in such work, they replied, “I think the overall motivation is that [the] Earth system science as a whole, including ocean and climate, is fundamentally societally relevant in terms of understanding the planet that we live on.” This researcher led a large research group, consisting of many PhDs, postdocs, and other permanent senior researchers. According to their CV and website, they had been awarded excellence grants (like an ERC Consolidator grant and a prestigious international ocean drilling research prize), and their work focused on fundamental questions of the past climate and climate proxy development, which allows scientists to more precisely know the past ocean’s characteristics, like temperature, salinity, acidity, and bioavailability of its nutrients.

This articulation aligns with an epistemic commitment that is dedicated similarly to *creating fundamental insights about the Earth System.* The articulation and corresponding epistemic commitment both attune to the excellence regime of worth, with prominent values and norms in contemporary academic culture such as competitiveness, peer recognition and productivity. Hence, while articulating the societal relevance of their work, proponents of this commitment at both institutions drew from these prominent and culturally common norms. Still, as we will show below, how the proponents of this articulation adhere to this epistemic commitment is dependent on their own positionality within epistemic living spaces, particularly in relation to their financial and cultural features.

Like the two articulations that follow, the fundamental articulation also corresponds with a certain conception of the science-society relation. Proponents of this articulation follow a linear model where science provides knowledge for society on its own terms and scientists share the fundamental knowledge they gained (i.e., mode one science), focusing on communicating the fundamental scientific knowledge gained to society to show its relevance to society. Despite the prevalence of this articulation, tensions may still arise as notions of doing societally relevant research shift. For example, this sentiment of relevance as communicating to society was brought up for debate in an internal meeting at institute A, when a researcher argued the institute needed to do more than communicate its scientific results if they were going to be considered “doing societally relevant research” for the next excellence funding round. However, this was not an opinion that was not shared by all, as another researcher lamented that they are “scientists, not communicators.”

Furthermore, society itself is viewed as one based on science and researchers tended to orient the worth their research will have for society towards some ambiguous future point and still unknown future applications. As one senior researcher, an organic geochemist from Institute A, elaborates:

We are living in a society that is based on knowledge. So, it's relevant to society, because science is relevant to society… we discover things that can be important [in the future]. People are interested in our planet, and we are contributing. We can see things that no one else has seen before. So, that's relevant for society to share this information.

The quote illustrates how importance is placed on the act of informing and, yet the “for whom” or “for what” remains vague; thus, creating a generalised notion of society that is envisioned at the receiving end of their communication efforts. The act of knowing more or making known to society is, thus, centred instead of direct connections to societal issues or specific communities. For example, while proponents of this articulation would seldom discuss their research in terms of its applicability to direct societal concerns, they often referred briefly to outreach, communicating their findings to a generalised public. When examining their CVs and institutional webpages, some researchers would have in the alternative outputs section talks on their area of interest either on the radio or written communications blogs, e.g. on “ocean heatwaves” or “what is the weather like in the deep sea?”. The focus here remains on communicating the fundamental scientific knowledge gained to society, with the media, thus, becoming a stand-in for “communicating to society” through which the researchers can communicate the relevance of their research to society.

### Two Subgroups of Articulation 1

The fundamental articulation of societal relevance was the most prevalent among the deep sea researchers interviewed. However, the positionality of the researcher within their respective epistemic living space had a notable influence on how they engaged in the necessary articulation work. Below, we demonstrate how the two subgroups of researchers, differing in their positionality in relation to their epistemic living spaces, adhere to the same fundamental articulation.

**The first subgroup of researchers** consists of researchers who credibly self-identified as excellent and required little articulation work to align with the fundamental articulation, which is in part due to the fundamental articulation’s strong alignment with the excellence regime of worth. Their claims of high performance, in comparison to their peers, line up with their examined research profiles (CVs and webpages), which contain conventional markers of excellence, e.g., high h-indexes, prestigious grants, international careers, professorships, and research group leadership, etc. Furthermore, when examining their research portfolios (through their CVs and online research profile, e.g., Google Scholar and institutional webpages), they concentrate on fundamental research lines (like developing new methods, exploring the deep biosphere, bettering the understanding of sediment transfer to the deep sea, etc.) and show little to no engagement with more applied or translational research lines, something that has stayed consistent through their academic careers. However, some have small mentions of science communication, like discussing their research on radio programs or writing outreach blogs. Additionally, they hold advisory positions on international boards in their respective fields, like on the scientific plenary committee for the coming period of the Ocean Drilling Program, but tend to not sit on scientific advisory committees geared toward informing policy.

We note that this ease of articulation was primarily present in Institute A. This finding aligns with the institute’s history of focusing on basic research lines, partially maintained by its consistent basic and excellence funding streams and reinforced by its excellence cluster on the deep sea environments. The institutional climate was conducive to this type of articulation for a long time. This history of institutional culture and valuing of excellence was observed not only in their previous strategy and website (which focused on finding fundamental insight through their excellence cluster) but also in interviews with its director and in institutional meetings. However, this mindset had more recently come under pressure due to their current re-application for another excellence cluster funding—their previous successful cluster application having received only one critique: the absence of societally relevant research lines. There was a sense that this articulation was not enough if they were to have a successful application to continue their excellence cluster. Still, the focus on excellence and fundamental knowledge as inherently relevant was quite prevalent. A group leader in paleoclimatology, the study of past climates, at Institute A shows this excellence mindset on fundamental knowledge:

“I think for most of us here [Institute A], we are still mostly doing fundamental research. And for that, the funders really are mostly interested in scientific excellence. And so, if you are internationally scientifically leading in the field, then the funders will fund you. And that route will not disappear, I don't think. I mean there, the funders will additionally call for, you know, perhaps societal relevance… So, those are aspects that drive a little bit how the actual funding bid say is formulated, but underlying all of that, that will still always be scientific excellence”

Lastly, exemplified by the latter half of the above statement by the group leader, many proponents of this articulation situate their research into relevance claims to match the funding calls but with the focus remaining on fundamental knowledge acquisition. Essentially, they retrofit their research portfolios. When asked about funding calls or societal impact sections (of grant proposals or project reports), they explained that their focus remains on continuing their existing research lines. They stressed the importance of making funding calls fit their research and do not engage in any re-orientation within or additions to their research lines, e.g., for ERC synergy grants or Horizon 2020 grants in the past.

**A second subgroup of researchers** that employed the fundamental articulation of relevance shared the same epistemic commitment to the creation of fundamental knowledge but were situated within very different epistemic living spaces. This difference in situatedness meant that maintaining alignment with this articulation and epistemic commitment necessitated different articulation work. Most notably they narrated a need to carve out places for themselves within academia. To do this, these researchers, unlike the previous subgroup, actively curated their research portfolios to create funding opportunities, while maintaining their fundamental research lines. Thus, their research portfolios could attune to multiple regimes of worth, e.g., excellence and societal relevance. A senior marine geologist from Institute B explains their motivation for starting more applied research lines and working on industry-funded projects: “I had to see that I got research funded which would pay my salary, here at [Institute B], so there was a direct motivation to do this.” This statement can be seen in their online research profile, as there is a distinct transformation over a short period of time (about two years), where deep sea mining starts to make up a substantial portion of their research output, where previously their work was around gaining fundamental knowledge about deep sea canyons, e.g., carbon cycling, nutrient cycles, and sediment flows. Unlike the articulations to follow, engaging with more societally relevant research lines becomes a means for continuing to research more fundamental questions within the epistemic living space they find themselves in.

While researchers within this subgroup similarly stress the value of and aligned with the excellence regime of worth, a prevailing norm in academic culture, they self-identify as outside of the career paths necessary to become a typical “excellent” researcher. They narrated this self-identification in multiple ways, from career breaks to alternative academic trajectories. Hence, what is valued remains similar (here, publication records) to the fundamental articulation, this subgroup’s researchers do not necessarily perceive themselves as high-performing in this regard, partially in relation to their peers.

For example, a professor in sedimentology, specialised in modelling, at Institute A told of rising to professor too quickly. So, while they held the position of professor, they spent a large portion of their time early in their career mentoring and organising (roles that were expected of them as a professor), which led to fewer publications than their peers who had larger postdoc career phases and were able to “focus more on themselves and building their careers.” Not having the publication record to apply for the big excellence grants (e.g., ERC grants), this researcher took on more roles in the institution, rising to leader of a focal area as well as taking on more applied research lines. For example, in addition to their more fundamental research on modelling deep sea sediment transport, this professor also engages in modelling sediment transfer in specific harbours for applied, directly relevant work.

### Working Towards Societal Relevance

Below, we will present two articulations that view the production of relevant knowledge not as something inherent to fundamental knowledge, but as a requiring further action to become relevant knowledge. How knowledge is made relevant differs based upon how the proponents of the articulations understand the relation between science and society and the norms to which they themselves subscribe. These actions differ from the articulation work of researchers presented in the preceding section, as it is seen as an essential part of making relevant knowledge. The two types of articulations observed were translational and solution-oriented.

### Articulation 2| Relevance as Translating Scientific Knowledge for Informing Policy

As the title suggests, the translational articulation of societal relevance focuses on creating and translating knowledge to inform policy decisions. While it values gaining a deeper understanding of the natural world (e.g., biodiversity in deep sea environments, nutrient cycling, sedimentation rates and flows, and past climates) its core emphasis is on making fundamental knowledge relevant and understandable for policy. Thus, researchers adhere to the epistemic commitment to *translate knowledge for decision makers*. Therefore, within this articulation fundamental knowledge alone is not inherently relevant and, instead, further action is needed—here the added translation by a scientist to become relevant knowledge.

Like the fundamental articulation, they also appeal to the excellence regime of worth, as they articulate their research as in alignment with prominent values in contemporary academia, like those of competitiveness, peer recognition, and productivity. However, researchers in this articulation additionally adhere to a second regime of worth: translational societal relevance, which values research in relation to its applicability for informing policy, often narrated in relation to pressing problems (e.g., deep sea mining, ocean acidification, ocean currents, and climate change).

While the researchers had different levels of engagement within policy realms, they would self-describe as experts on the topics they research. Their research profiles show a history of a duality (when examining their CVs and webpages) of excellence in a specific topic (i.e., international careers, group leaders, high h-indexes) and of policy work, e.g., membership to different policy advisement boards (for example the European Marine Board (EMB) working group or the International Seabed Authority (ISA)), committees, presentations, or authorship on policy documents. This combination of their research lines and policy activities was also apparent in their research portfolios. The researchers in this articulation narrated the curations of their research portfolios to include both fundamental and societally relevant research lines, which was echoed in their CVs and online profiles. This curation sometimes would lead to more projects that would inform policy.

For example, one senior researcher from Institute B, who is heavily involved in gaining more knowledge about deep sea benthic biology to better inform policy about possible impacts on deep sea environments, acquired funding for testing man-made deep sea nodules as a replacement for the ones extracted by industry. As they explained in their talk at a deep sea biology symposium, it has long been claimed by industry actors to deep sea mining regulators that these nodules (which take thousands if not millions of years to regrow) could not be replaced to preserve environments for deep sea organisms who are dependent on them. However, no one had tested this theory, so policies on deep sea mining that would allow for such replacements could not be formulated one way or the other. This lack made them want to test out this hypothesis to see if deep sea organisms would even grow in such conditions and if so, on which substrates, so they found funding for a five year long pilot project.

Proponents of this articulation and commitment view society as in a state of multiple crises, which need to be addressed through policies created by governing bodies. Science’s role is to provide enough information such that policymakers can make informed decisions. Yet oftentimes, they say, science does not know enough. They therefore locate the worth of their research for society in the present, and they directly conflate society with policy realms. Still, the role of science aligns with traditional scientific norms of objectivity and impartiality, as exemplified in this quote by a physical oceanography professor, working on ocean acidification, from Institute B: “we inform, it is very important that we are independent. I have of course opinions about these kinds of things, private [ones], but it is our role in society to provide information.” This quote is an example of how traditional scientific norms of disinterestedness are upheld. While they do not completely align with a linear model, or mode one science, proponents of this articulation still see science’s role as one outside of society, as a provider of information for policy to make informed decisions, but not as an active contributor in providing solutions.

The translational articulation was observed almost entirely at Institute B. The prevalence of this articulation seems to be tightly connected to institutional culture as well as the institution’s position within its broader national context—and less connected to individual choice. Institute A was still in a transformative phase, only recently moving to incorporate new ways of fostering and doing societally relevant research (a long process witnessed during our observations of their internal meetings). Their director explained they did not encourage their researchers to engage with policy previously and are now finding it more difficult as an institute to foster such engagements. For example, Institute A was not a member of the European Marine Board, the leading European think tank in ocean policy, while Institute B was. Institute B, conversely, has a long history of engaging with ocean policy, as the main ocean research centre for the country. As the director explained in an interview, Institute B’s focus is on fundamental research, as there are other ocean research institutes in the country that perform strictly applied research (e.g., contract work for industry and environmental monitoring for governmental assessment). This combination of valuing fundamental research and excellence, while simultaneously feeling of responsibility to inform policy is conducive for translational work. In fact, one interlocutor within the institute’s management team prided themselves that the analysis of the institute’s academic outputs for a research assessment exercise showed that the institute had doubled their impact in policy documents. They saw this as “suggesting that a lot of our papers from the overall papers that we publish, a lot of them are used in policy documents.”

### Articulation 3| Relevance as Providing Solutions for Societal Issues

The solution-oriented articulation centres societal relevance as *the creation of directed knowledge*; thus, societally relevant research is narrated as directed research that takes an active role in developing potential solutions to the multitude of crises facing society, e.g., carbon storage, carbon emissions reduction, incorporation of green technologies in ocean environments, climate mitigation, and mitigating the damaging effects of deep sea mining. In this articulation, research only comes to be understood as relevant through the process of directing knowledge. Unlike the previous articulations, fundamental knowledge is de-centred by aiming to produce actionable knowledge from the onset of the research process. Societal relevance is understood as a quality criterion for their research and its corresponding knowledge creation.

This articulation aligns with the epistemic commitment dedicated to the creation of *directed knowledge for solutions to urgent environmental problems facing society*. Most interestingly, proponents of this commitment seem to challenge the excellence regime, by questioning the worth of their fundamental research in a world in crisis. Alternatively, they draw from a regime of worth focused on the direct relevance of research. They cite an urgent need to do something for society, particularly in relation to the climate crisis. They usually fund these activities through grants that focus on economic valorisation and collaboration with societal actors, e.g., a national Dutch grant that required results to be “embedded in society” or a Horizon 2020 grant led by a mining company. The narratives of the worth of their research centre on their ability to directly act in solution-making, and often take on a more personal tone as the researchers strive to find solutions to problems that matter to them (like carbon sequestering). As one professor in petrology from Institute A laments:

Dogmatism [here: about researching the environmental problems and their effects, i.e. climate change] really doesn't help us and if you don't take action, then, you know, you can still do research, but you're just observing disaster happening.

This quote shows both the researcher’s frustration with more traditional ways of doing science, e.g., disinterestedness and impartiality, as well as their personal drive for engaging in research that takes more action. This directness of the research presents itself in the articulation as specific problems to be solved and specific communities to be helped. For example, one senior biogeochemist from Institute A was getting involved in farming kelp in the ocean to extract large amounts of carbon from the atmosphere, while also helping feed communities suffering from food scarcity. The project was a collaboration between scientists studying both the effects of the kelp farm on the local environment, e.g., nutrient cycles and species distribution, as well as the amount of carbon stored in the kelp and an industry partner wanting to start kelp farming in the deeper waters off the coast of an African nation.

While researchers adhering to this articulation had various career paths, some would also self-identify as high-performers, and their CVs and online profiles confirmed that they had research profiles to match. However, unlike those who easily aligned with the fundamental articulation, researchers did not equate as much value to publication and citation records, at least at the current stage of their career. Oftentimes, they narrated how this devaluing of fundamental, excellent research developed as they came to understand and realise the climate crisis. As one senior scientist in geochemistry from Institute A explains, in reference to their fundamental research on the deep ocean seafloor:

At the end, you can say that it's unique, that you see it for the first time and so on. But then, you can ask, yeah, so what? Okay, oxygen consumption at 8000 meters deep is higher than we assumed. Oh, wonderful, yeah? But only two per cent of the ocean seafloor are trenches. You make a record, but in principle you can say, fuck off! For what? Yeah, now we know this, wonderful, nice but what can we learn from this?

In this quote, they devalued their previous work on deep sea trenches, because they started to feel that the knowledge gained, however fascinating, was detached from society. Although they still conducted fundamental research on the deep biosphere and the roles of various elements in the nutrient cycles there, they also took up more directly applicable research lines. For example, they had just become PI of a subproject of a larger project (funded by a German national grant) on carbon storage, which focuses on researching innovative technologies and approaches for monitoring sub-seabed storage of carbon. This valuing of directly applicable research lines led researchers in this articulation to also engage in curating their research portfolios, so that they were cultivating both more fundamental and more solution-oriented research lines. They expressed feeling a sense of duty to help by providing solutions to the climate and environmental crises with their research and discussed actively seeking opportunities to expand their research portfolios to take part in researching potential solutions to problems like carbon sequestering or ocean acidification. Their CV and online research profiles matched their narrations of their research portfolios and contained, to varying degrees, a mixture of fundamental and applied research lines and corresponding projects, oftentimes with a visible increase towards more directed relevant research.

This sense of duty to help also carried over into how they view science-society relations. They narrated society as specified communities that could be helped. However, when speaking of climate change more broadly, society was often conflated with a general mankind of which the researchers were a part. Still, in their science communications researchers in this articulation tended to engage more strongly and opinionatedly with society. For example, giving more interviews for radio and tv in which they share their opinions more freely, e.g., taking part in a documentary problematising deep sea mining. They see their role as one that needs to interact more with society and some take action to do so, e.g., by helping to organise social dialogue events on carbon storage. Some of the interlocutors were active participants in climate protest organisations and were actively protesting science funding by oil companies.

The solution-oriented articulation was present in both institutional contexts, with slight differences between the profiles of the researchers depending on the institute. Institute B researchers often tended to also be involved in translational policy work, while Institute A researchers tended towards solution-oriented relevance research alone. This difference can probably be attributed to the institutional context of the researchers, with Institute B having more of an institutional commitment to engaging in translating knowledge for policy work. As mentioned above, Institute A, on the other hand, previously had no such commitment. Thus, individuals from Institute A, adhering to the solution-oriented articulation, engage from a personal, individualistic standpoint (potentially from a more value-driven place) rather than one that has been historically more institutionally framed.

## Discussion

This article explored three articulations of societal relevance that deep sea researchers align with when narrating the worth of their research in relation to society within their epistemic living spaces. We showed how researchers’ adherences to certain epistemic commitments within the ecology of their epistemic living spaces shaped how they articulated the relevance of their research, while simultaneously demonstrating that the actions taken to stay in alignment with these articulations are dependent on and informed by the epistemic living spaces inhabited by the researchers.

We presented the articulations as idealisations based on two major distinctions. The first distinction was identified between the types of knowledge the researchers understand as relevant for society and how this relevance comes to be. This distinction separated the fundamental articulation from the translational and solution-oriented articulations, whom both see additional actions as necessary for creating relevant knowledge. The fundamental articulation understood fundamental knowledge as inherently relevant. It was defined by the epistemic commitment to fundamental knowledge production and a strong alignment to the excellence regime of worth. The discussion of their results within media outlets served as a stand-in for communicating with society. However, we have shown how positionality in one’s epistemic living space matters for how researchers aligned with this articulation and the types of articulation work required (e.g., research portfolio curation).

While all researchers did engage in some sort of fundamental knowledge production in their research portfolios, researchers in the other two articulations we observed understood the production of relevant knowledge not as something inherent to fundamental knowledge, but instead as requiring further action. How this is done differs depending on how the proponents of the articulations understand the relation between science and society, the norms to which they themselves subscribe, and their epistemic living spaces. Our second articulation centred around the creation and translation of knowledge to inform policy decisions, and its researchers shared the epistemic commitment to translating knowledge for decision-makers. The researchers in this articulation draw from two regimes of worth simultaneously: the dominant excellence regime of worth and the regime that values translational societal relevance. Proponents see science’s role as one of informing society and—without fully adhering to mode one science—still see science as outside of society, as a provider of pertinent information for policy decisions, not as a provider of solutions. This articulation appeared to be dependent on the institutional feature of researchers’ epistemic living spaces, particularly in relation to institutional culture and its broader national context.

Our third articulation centred around societal relevance as the creation of directed knowledge. Its proponents adhered to the corresponding epistemic commitment dedicated to the creation of directed knowledge for solutions to urgent environmental problems facing society. In contrast to the previous two articulations, this articulation de-centres fundamental knowledge and instead, aims to create actionable knowledge throughout the research process, making societal relevance into a quality criterion for their research and its corresponding knowledge creation. Proponents of this articulation have a sense of duty to help which carries over into how they view science-society relations. Accordingly, society is narrated as specific communities that could be helped. In their science communications researchers in this articulation tended to engage stronger and more opinionatedly with society.

As mentioned previously, these three identified articulations are idealisations to help tease out patterns amongst the inherent real-world messiness of epistemic living spaces. Part of this observed messiness arose from researchers who were in the process of changing their articulations, e.g., from a more fundamental articulation to a solution-oriented one. One researcher we talked to from Institute B became engaged with deep sea mining as a means of continuing their fundamental research career, but through the engagement in projects with industry, they gradually started to value the work in its own right. It was through their involvement in the projects with industry that they began to see the importance of such engagement and the responsibility they have as scientists to society and the environment. A second example was observed in a senior researcher at Institute A who was very focused on their fundamental research and reflected that throughout their career they preferred fundamental research without an immediate application, but now that they have begun thinking more about climate change and how to help, they want to learn how to translate their knowledge. This shift, while still incipient, shows that articulations are not static.

Another important observation was how the articulations related to the knowledge creation process and what was understood to be “good” knowledge. The fundamental articulation subscribed to a linear model of knowledge production, wherein science produces knowledge that society can uptake thereafter but is not involved in the shaping of it. The translational articulation took a slightly altered view of the science-society relation regarding knowledge production. However, it still understood science’s role in a more traditional sense, as disinterested and separate from society. Only in the solution-oriented articulation did societal relevance become a criterion for knowledge creation, fully integrating relevance in the knowledge production process, adhering to a more mode two knowledge production process (Nowotny et al. 2001). These understandings affected the types of problems addressed and knowledge created. For example, in the translational articulation, knowledge was created around phenomena like climate change, ocean acidification, and learning more about deep sea environments, while in the solution-oriented articulation knowledge is directed at more practical solutions, e.g., ocean carbon sequestering, green technologies, damage mitigation.

## Conclusions

The field of deep sea research is in transition regarding its understanding of societally relevant research. Historically more focused on fundamental knowledge production and traditional value narratives such as novelty, the field is now criticised for failing to demonstrate the relevance of the deep sea for society (Jamieson et al. 2021) and for creating a disconnect between epistemic foci and concerns of scientific researchers and industry (Rogers et al. 2015). The positioning of deep sea research as a field actively experiencing this intensification of valuing societal relevance, differs from previous studies of relevance articulation in STS (see Granjou and Arpin 2015; Sigl et al. 2023). These studies have explored fields that since their inception have oriented themselves towards societal and environmental concerns. As calls for societally relevant research are intensifying, we have shown that it is also important to explore fields in transition, such as deep sea research.

Our research further demonstrates that shifts towards new articulations of societal relevance are not only observable at the field level (c.f. Granjou and Arpin 2015; Sigl et al. 2023) but also occur on a smaller scale, between and within institutes, a phenomenon understudied in STS thus far. Our analysis of individuals’ epistemic living spaces (Felt 2009; Felt and Fochler 2012) and our person-centred approach aids in teasing out the specific contexts, values, and valuations from which researchers draw whilst articulating the value of their research. Our focus on epistemic living spaces testifies to the importance of studying how the various actors of the science system (e.g., funders, research institutions, journals, etc.) play a role in understanding and articulating relevance at all scales, as they all influence how researchers can and do think about doing relevance within their epistemic living spaces. This multiplicity of influential actors at multiple scales necessitates not only multiple policy initiatives but also conversations between different actors across the science system around what doing relevance can mean substantially.

We focused our analysis on four contributing features to epistemic living spaces (institutional context, financial and evaluative factors, cultural norms, and mediatisation), which we observed playing a crucial role in shaping both the researchers’ epistemic living spaces and their articulations of relevance. The institutional dimension provided framing narratives around what relevance means, while the position of the institution within the larger national context fostered certain understandings of the role of science in society. This suggests that institutions have a key role and responsibility in developing framework narratives for doing relevance to which researchers can relate. Additionally, cultural norms played a role in shaping relevance articulations, particularly with respect to how they were embedded within the institution, which often (un)favoured certain regimes of valuation (e.g., the excellence regime) while fostering adherence to certain epistemic commitments. Thus, institutional narratives of relevance also need to be reflected in cultural values and quality criteria within institutions, to avoid producing the typical tensions between “talk” and “what actually counts” as relevance.

The financial dimension was an important factor to researchers and our results show that coupling funding to expectations of relevance is effective in incentivising researchers to articulate the relevance of their work. Yet, our results also demonstrate that these articulations may occur in very different ways—some of which can be perceived as rather rhetoric and some as more substantive, with reactions of researchers to funding incentives depending on their adherence to certain epistemic commitments. Lastly, we demonstrated the role of one’s perception of science’s relation to society plays in understanding relevance, which was originally conceptualised as mediatisation by Felt and Fochler (2012). However, their notion of mediatisation might be too limiting for capturing the dynamics of the science and society relation that played such an important role in shaping relevance articulations. Instead, perhaps, thinking of a factor that incorporates communicating and interacting with society more broadly might be more apt for understanding present-day science-society relations and how they shape epistemic livings spaces. This broadening of conceptualisation would create space for considering aspects such as engaging with industry, working with stakeholders’ groups, or even participating in and speaking at climate protests.

Our findings demonstrate the diversity of factors that shape how deep sea researchers understand and articulate the worth of their research in relation to society as well as in the understanding of relevance itself. This resonates with the practice-based approach to understanding relevance proposed by the editors of this special issue (see their introduction). The diversity we find highlights the importance for actors in research governance to reflect upon what “doing relevance” really means in terms of how knowledge is both produced and becomes relevant for society. Furthermore, these articulations facilitate discussions around diversity in understandings of relevance, wherein even commitments to fundamental research can be brought into the discussion, without the reductive stance often occurring of “everything is relevant.” Our ideal type categorisations of relevance articulations offer a starting point for such discussions and reflections, but certainly, these types will need specification and adaptation to every context. Still, articulations create specific language for productive dialogues about relevance. They may also be of use for funders or policy actors aiming to support certain types of research, allowing them to direct their calls more precisely towards fundamental, transitional, or solution-oriented logics. However, this language of relevance articulation is particularly important considering our findings on funding drivers. Variations in the fundamental articulation demonstrate that the absence of a personal driver may lead to retrofitting research lines into relevance claims and to cosmetic practices in strategic portfolio building. Thus, completely top-down governance measures towards societal relevance will mostly be insufficient or potentially incentivise strategic adoption rather than multi-level substantive change.

Of course, to have such discussions around diversity in notions of relevance warrants reflections on definitions of relevance. Our case of deep sea research, a field in transition that is currently developing narratives around how societal relevance is created, offered a unique chance to understand relevance and relevance creation as a process, particularly as institutes themselves adjust to this intensification of calls for societally relevant research. To achieve this, we started from our proposed definition of relevance, as *the perceived worth one’s research can acquire in relation to society.* This broadens and moves beyond Hessels et al.’s (2009, 388) definition of the term as “the expected value scientific research will have for society.” With this expanded definition, attention is placed on the researchers’ perceptions of relevance as an active process, situating relevance claims in the present rather than as future outcomes or impacts. This revised definition may also offer new ways of discussing and evaluating relevance claims beyond the necessarily uncertain character of their projected futures. In doing so, however, acknowledging the inherent multiplicity of the term without committing to a singular *a priori* normative notion of “doing relevant research” remains central.

## Footnotes

1 This paper is part of the special issue “Modes of Relevance in Research. Towards Understanding the Promises and Possibilities of Doing Relevance“.

2 It is important to note that substantial portions of research on the ocean remain privately funded. This private funding comes from two main sources: military and industrial interests (Oreskes 2021b), and philanthropic institutes. Examples of the latter include the Schmidt Ocean Institute, OceanX, and The Nippon Foundation’s Ocean Nexus.

3 For a more detailed explanation of the history and diversification of the term relevance, please refer to the introduction of this special issue.
